# Blockade of β-Adrenergic Receptors by Propranolol Disrupts Reconsolidation of Drug Memory and Attenuates Heroin Seeking

**DOI:** 10.3389/fphar.2021.686845

**Published:** 2021-05-25

**Authors:** Liangpei Chen, Shihao Huang, Chang Yang, Feilong Wu, Qiuyao Zheng, He Yan, Jie Yan, Yixiao Luo, Ewa Galaj

**Affiliations:** ^1^ Key Laboratory of Molecular Epidemiology of Hunan Province, School of Medicine, Hunan Normal University, Changsha, China; ^2^ Department of Forensic Science, School of Basic Medical Science, Central South University, Changsha, China; ^3^ National Institute on Drug Abuse, Molecular Targets and Medications Discovery Branch, Baltimore, MD, United States

**Keywords:** drug memory, propranolol, reconsolidation, relapse, heroin, drug seeking, reinstatement

## Abstract

Persistent traces of drug reward memories contribute to intense craving and often trigger relapse. A number of pharmacological interventions on drug-associated memories have shown significant benefits in relapse prevention at a preclinical level but their translational potential is limited due to deleterious side effects. Propranolol, a non-specific β-adrenergic receptors antagonist, is known for its ability to erase maladaptive memories associated with nicotine or cocaine in rodents and humans. However, little is known about its effect on reconsolidation of heroin memory and heroin seeking. In the present study, rats with a history of intravenous heroin self-administration received the propranolol treatment (10 mg/kg; i.p.) at different time windows with or without CS (conditioned stimulus) exposure. Our results showed that propranolol, when administered immediately after CS exposure but not 6 h later, can significantly attenuate cue-induced and drug-primed reinstatement of heroin seeking, suggesting that propranolol has the ability to disrupt heroin memory and reduce relapse. The propranolol treatment without retrieval of drug memory had no effect on subsequent reinstatement of heroin seeking, suggesting that its interfering effects are retrieval-dependent. Importantly, the effects of propranolol were long lasting as rats showed diminished drug seeking even 28 days after the treatment. Altogether, our study suggests that propranolol can interfere with reconsolidation of heroin memory and reduce subsequent drug seeking, making it an attractive therapeutic candidate for the treatment of opioid addiction and relapse prevention.

## Introduction

Opioid use disorder, a chronic and recurrent brain disease (Leshner, 1997; Volkow et al., 2016), is considered as a maladaptive learning and memory disorder (Boening, 2001; Hyman, 2005). Much evidence shows that craving, compulsive opioid taking and seeking in human addicts are controlled by drug cues and opioid reward-associated memories (Childress et al., 1988; O’brien et al., 1992). In animal models of addiction, opiate-associated cues can reinforce intravenous drug self-administration (Davis and Smith, 1976; Dymshitz and Lieblich, 1987; Di Ciano and Everitt, 2004), facilitate the acquisition of opiate tolerance (Siegel, 1975), enhance locomotor activity (Mucha et al., 1981), elicit conditioned place preference (Schenk et al., 1983; Bardo et al., 1984; Bardo and Neisewander, 1986) and reinstate drug seeking even after prolonged abstinence (Schuster and Woods, 1968; McFarland and Ettenberg, 1997; Peck and Ranaldi, 2014). Thus, the persistence of drug memories and difficulty in eliminating them are thought to be the root causes of compulsive drug use, seeking and relapse (Childress et al., 1986; Kelley, 2004; Torregrossa et al., 2011).

A number of pharmacological interventions on drug-associated memories have shown significant benefits in relapse prevention at a preclinical level but their translational potential is limited due to deleterious side effects that the amnestic agents produce (Lee et al., 2005; Milton et al., 2008a; Milton et al., 2008b; Taylor et al., 2009; Sanchez et al., 2010; Wells et al., 2013). Drug memories can become labile after retrieval (Lee, 2009; Phelps and Hofmann, 2019), and require protein synthesis to be re-stabilized. During this process, known as memory reconsolidation, labile drug memories can be manipulated by pharmacological agents such as U0126 (an inhibitor of the mitogen-activated protein kinase1/2) or anisomycin (a peptidyl transferase inhibitor), leading to a reduction in subsequent cocaine- or nicotine-conditioned place preference (CPP) and drug seeking (Miller and Marshall, 2005; Lv et al., 2015; Sorg et al., 2015; Xue et al., 2017a). However, clinical applications of these pharmacological agents are fairly limited due to their side effects.

Propranolol, a non-specific β-adrenergic receptor (β-AR) antagonist, appears to be a promising candidate. Emerging evidence from clinical and preclinical studies reveals that noradrenergic signaling plays a critical role in memory reconsolidation (Schwabe et al., 2012; Haubrich et al., 2020). Unlike U0126 or anisomycin, propranolol can be safely administered to humans and effectively reduce cocaine or nicotine craving in human population (Saladin et al., 2013). In animal models of drug abuse, propranolol attenuates cocaine, morphine and nicotine seeking CPP through disruption of the association between drug cues and drug rewarding effects (Bernardi et al., 2006; Robinson et al., 2011a; Xue et al., 2017b). However, to date, there is no direct evidence indicating that propranolol can reduce heroin craving and prevent relapse. Given that heroin addicts show abnormal functioning of alpha2-adrenoceptors in the brain (Meana et al., 2000) and that the noradrenergic system is involved in drug memory reconsolidation (Fricks-Gleason and Marshall, 2008; Otis et al., 2015), it is conceivable that propranolol might disrupt reconsolidation of heroin memory and attenuate craving for heroin. If so, one would expect that propranolol would diminish cue- and drug-induced reinstatement of heroin seeking. Thus, in this study, we assessed whether propranolol can interfere with memory reconsolidation when administered at different time windows with or without CS (conditioned stimulus) exposure and whether its effects on heroin seeking are long lasting.

## Materials and Methods

### Subjects

Male Sprague Dawley rats (weighing 260–280 g on arrival) were housed in groups of five under a 12 h reversed light/dark cycle (lights off at 8:00 A.M. and lights on at 8:00 P.M.) in a climate-controlled environment with a constant temperature (23 ± 2°C), humidity (approximately 60%), and with free access to food and water. Prior to surgeries, rats were handled for 3 min per day for five consecutive days. Animal care and experimental procedures were conducted in accordance with the National Research Council and Hunan Province Guide for the Care and Use of Laboratory Animals. All experiments were approved by the Biomedical Ethics Committee for Animal Use and Protection of Hunan Normal University. The experiments were performed during animals’ active cycle (i.e., dark cycle).

### Intravenous Surgery

Rats (weighing 300–320 g at the time of surgery) were anesthetized with sodium pentobarbital (60 mg/kg, i.p.). Catheters were inserted into the right jugular vein with the tip terminating at the opening of the right atrium as described previously (Lu et al., 2005; Ambroggi et al., 2008). The catheter was connected to a bent 22-gauge stainless steel connector mounted to the rat’s skull using four stainless steel screws and dental acrylic. The intravenous catheters were kept patent by infusion of 0.1 ml of heparinized saline (30 USP heparin/saline; Hospira) every 2 days. Before the start of experimentation rats were allowed to recover for 5–7 days.

### Behavioral Procedures

#### Intravenous Heroin Self-Administration Training

The heroin self-administration training paradigm and conditions were adapted from our previous studies (Ye et al., 2017). Operant chambers (AniLab Software and Instruments, Ningbo, China) were equipped with two nosepoke operandi (AniLab Software and Instruments, Ningbo, China) located 5 cm above the floor and with light stimuli. Rats were trained to self-administer heroin intravenously (0.05 mg/kg/infusion) during three 1-h daily training sessions separated by 5 min for 10 days. We used a fixed-ratio 1 (FR1) schedule of reinforcement with a 40-s time-out employed after each infusion. Briefly, rats were connected to a drug line consisting of a metal tether covering a polyethylene tubing which, through a fluid swivel (Instech, Plymouth Meeting, PA), was connected to a syringe pump loaded with a 10 ml syringe. The session began with the illumination of a house light that remained on for the entire session. Nosepokes into the active operandum led to a delivery of intravenous heroin accompanied by presentation of a 5-s tone-light cue. Nosepokes into the inactive operandum were counted but had no consequences. The heroin self-administration procedure was used in all four experiments. We excluded a total of eight rats from the experiments: three rats due to catheter patency failure and five rats due to failure to acquire heroin self-administration.

#### Nosepoke Extinction

Following the drug self-administration phase, rats were subjected to the extinction training. During 3-h daily nosepoke extinction sessions (Experiments 1–4), nosepokes to either of the operandi had no programmed consequences (i.e., heroin infusion and conditioned tone-light cues were not delivered). Rats were subjected to the extinction training until the frequency of active nosepoke responding decreased below 20% of the average responding during the last three heroin self-administration sessions for at least two consecutive days.

#### Reactivation of Heroin Memory

A 15-min session to reactive heroin-associated memories commenced 24 h later after the last nosepoke extinction session (in Experiment 1, 2, 4). The retrieval conditions were the same as during the heroin self-administration training except that active nosepokes were reinforced with drug cues but not heroin.

#### Cue Extinction

During 3-h daily cue extinction sessions (Experiments 1,3,4), the conditions were the same as during the heroin self-administration training, but no heroin infusions followed the delivery of cue (tone/light).

#### Propranolol Treatment

Rats were injected with saline or propranolol (i.p. 10 mg/kg) immediately after the CS exposure (reactivation session) (Experiments 1, 2). In Experiment 3, rats received the propranolol or saline treatment without CS exposure and in Experiment 4, 6 h after the CS exposure rats received an intraperitoneal injection of propranolol or saline and were housed in their home cages until further testing.

#### Cue-Induced Reinstatement of Drug Seeking (Experiments 1–4)

Twenty-four hours after the propranolol or saline treatment (i.p.), rats were subjected to the reinstatement test induced by drug cues. The test condition was identical to that of heroin self-administration training, with the exception that active nosepokes led to contingent presentations of the tone-light cue that had been previously paired with heroin and were not reinforced with heroin. The reinstatement test lasted 1 h.

#### Heroin-Induced Reinstatement of Drug Seeking (Experiments 1, 3, 4)

Rats were injected with heroin (0.25 mg/kg, i.p.) 5 min before being placed into the self-administration context. The test conditions were the same as that of the drug self-administration training with the exception that active nosepokes were reinforced with drug cues but not heroin. The reinstatement test lasted 1 h.

#### Cue-Induced Reinstatement Test After Prolonged Withdrawal (Experiment 2)

For the cue-induced reinstatement test after prolonged withdrawal, active and inactive operandum nosepokes were recorded for 1 h 28 days after the forced withdrawal. The testing conditions were the same as those during the cue-induced reinstatement test.

### Specific Experiments

#### Experiment 1A and B: The Effect of Immediate Post-Conditioned Stimulus Reactivation Propranolol Treatment on Subsequent Cue-Induced and Drug-Primed Reinstatement of Heroin Seeking

**FIGURE 1:**
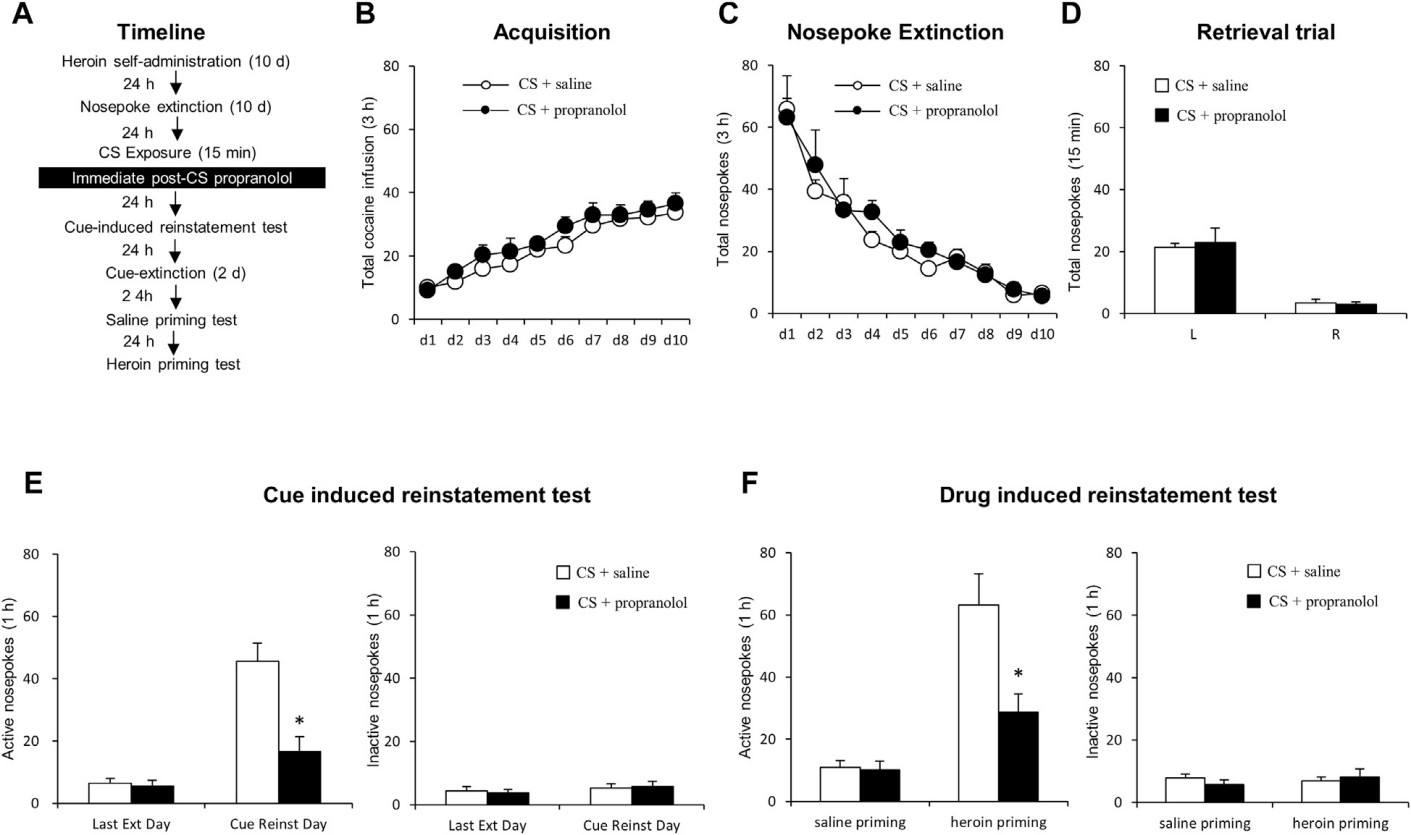
Immediate post-CS reactivation propranolol treatment reduces subsequent cue-induced and heroin-primed reinstatement of heroin seeking. **(A)** Schematic representation of the experimental procedure. **(B)** Total number of heroin infusions across acquisition of heroin self-administration. **(C)** Total number of active nosepoke responses across extinction sessions. **(D)** Nosepoke responses during a reactivation trial. **(E)** Active **(left)** and inactive **(right)** nosepoke responses during the last extinction session and the cue-induced reinstatement test. **(F)** Active **(left)** and inactive **(right)** nosepoke responses during the saline- or heroin-primed reinstatement test.

Rats received heroin self-administration training for 10 days, followed by nosepoke extinction training for 10 consecutive days in the same operant chambers. Twenty-four hours after the last nosepoke extinction session, rats received a 15-min CS reactivation session induced by re-exposure to the heroin training context. Immediately after the CS reactivation session rats received an intraperitoneal injection of propranolol or physiological saline and 24 h later, a cue-induced reinstatement test was performed to verify whether the administration of propranolol immediately after retrieval of heroin cue memory destroys the expression of heroin cue memory in rats. Twenty-four hours after the cue-induced reinstatement test, rats received daily cue extinction sessions for two consecutive days. Twenty-four hours later, rats were tested for saline priming-induced reinstatement. Twenty-four hours after the saline priming-induced reinstatement, rats received heroin-induced reinstatement test. (see Figure 1A).

#### Experiment 2: The Effect of Acute Treatment With Propranolol on Cue-Induced Reinstatement and Spontaneous Recovery 28 days Later

In this experiment, rats received the propranolol treatment immediately after 15-min CS reactivation trial. Twenty-four hours later, a cue-induced reinstatement test occurred, followed by 28 days’ abstinence and another cue-induced reinstatement test.

#### Experiments 3 A and B: The Effect of the Propranolol Treatment Without Conditioned Stimulus Reactivation on Subsequent Cue-Induced and Drug-Primed Reinstatement of Heroin Seeking

The experimental procedure was identical to that of Experiment 1, except that rats received an intraperitoneal injection of propranolol immediately after a 15-min no-reactivation session (see Figure 3A).

#### Experiment 4 A and B: The Effect of Delayed Post-Conditioned Stimulus Reactivation Propranolol Treatment on Subsequent Cue-Induced and Heroin Primed Reinstatement of Drug Seeking

The experimental procedure for Experiment 4 was identical to that of Experiment 1, except that the rats received an intraperitoneal injection of propranolol 6 h after a 15-min reactivation session (see Figure 4A).

### Statistical Analysis

Experimental results were presented as mean ± SEM and analyzed by GraphPad, v.8.0. The data were analyzed by repeated measures ANOVAs with between-subjects factor of treatment condition and within-subjects factor of test condition followed by Tukey’s *post-hoc* test in each experiment (see results). *p* < 0.05 were considered statistically significant.

## Results

### Experiment 1: Immediate Post-Conditioned Stimulus Reactivation Treatment With Propranolol Reduces Subsequent Reinstatement of Heroin Seeking

We employed two groups of rats to test the effect of the post-reactivation propranolol treatment on cue- and heroin-induced reinstatement of heroin seeking (Figure 1A). Groups-to-be treated with propranolol (*N* = 9) or saline (*N* = 9) did not differ during the acquisition of heroin self-administration, as indicated by the similar total numbers of heroin infusions [main effect of the training day: F _(9,144)_ = 23.906, *p* < 0.001; main effect of the treatment condition: F _(1,16)_ = 4.092, *p* = 0.060; interaction of training day × treatment condition: F _(9,144)_ = 0.286, *p* = 0.978; Figure 1B]. No significant group differences were found during extinction, as revealed by the similar numbers of active and inactive nosepokes over 10-day extinction sessions [main effect of the extinction day: F _(9, 144)_ = 28.961, *p* < 0.001; main effect of the treatment condition: F _(1,16)_ = 0.690, *p* = 0.418; interaction of extinction day × treatment condition: F _(9, 144)_ = 0.443, *p* = 0.901; Figure 1C]. For the reactivation test, there were no group difference of heroin infusions during the retrieval test [main effect of the different nosepokes: F _(1,16)_ = 69.05, *p* < 0.001; main effect of the treatment condition: F _(1,16)_ = 0.062, *p* = 0.807; interaction of different nosepokes × treatment condition: F _(1,16)_ = 0.214, *p* = 0.650; Figure 1D].

A two-way repeated-measures ANOVA for the cue-induced reinstatement data revealed a significant group difference in active nosepokes during the reinstatement test [main effect of the test condition: F _(1, 16)_ = 34.84, *p* < 0.001; main effect of the treatment condition: F _(1,16)_ = 20.82, *p* < 0.001; interaction of test condition × treatment condition: F _(1,16)_ = 10.97, *p* = 0.004; ]; *Post-hoc* shown that drug-seeking in the Retrieval + Propranolol group was significantly reduced as compared to the Retrieval + Saline group in the cue-induced reinstatement test (*p* < 0.05) (Figure 1E left column), but not in inactive side nosepokes [main effect of the test condition: F _(1,16)_ = 1.083, *p* = 0.314; main effect of the treatment condition: F _(1,16)_ = 0.008, *p* = 0.931; interaction of test condition × treatment condition: F _(1,16)_ = 0.103, *p* = 0.753; Figure 1E right column]. In addition, there was a significant group difference in active nosepokes during the heroin-primed reinstatement test [main effect of test condition: F _(1,16)_ = 44.53, *p* < 0.001; main effect of treatment condition: F _(1,16)_ = 8.31, *p* = 0.011; test condition interaction × treatment condition: F _(1,16)_ = 10.08, *p* = 0.006];A *Post-hoc test* revealed that drug-seeking in the Retrieval + Propranolol group was significantly reduced compared to the Retrieval + Saline group in the priming-induced reinstatement test (*p* < 0.05) (Figure 1F left column), but not in inactive nosepokes [main effect of the test condition: F _(1,16)_ = 0.251, *p* = 0.623; main effect of the treatment condition: F _(1,16)_ = 0.058, *p* = 0.813; interaction of test condition × treatment condition: F _(1,16)_ = 1.154, *p* = 0.299; Figure 1F right column]. The results of this experiment indicate that intraperitoneal injection of propranolol immediately after the heroin cue reactivation suppresses cue-induced and heroin-primed reinstatement of heroin seeking.

### Experiment 2: Immediate Propranolol Treatment Following Conditioned Stimulus Reactivation has Long Lasting Attenuating Effects on Heroin Seeking

**FIGURE 2:**
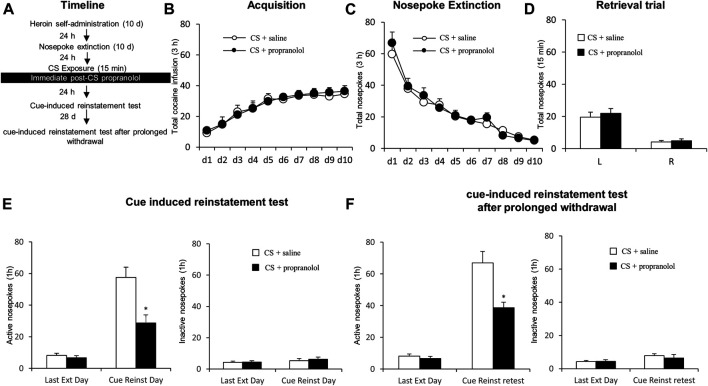
Propranolol treatment immediately after CS reactivation attenuates subsequent and post-abstinence cue-induced reinstatement of heroin seeking. **(A)** Schematic representation of the experimental procedure. **(B)** Total number of infusions across heroin self-administration sessions. **(C)** Total number of active nosepoke responses across extinction sessions. **(D)** Nosepoke responses during a reactivation trial. **(E)** Active **(left)** and inactive **(right)** nosepokes during the last extinction session and cue-induced reinstatement test. **(F)** Active **(left)** and inactive **(right)** nosepokes during the last extinction session and cue-induced reinstatement test after prolonged withdrawal (after 28 days of abstinence).

We aimed to assess the effect of immediate post-CS propranolol injection on cue-induced reinstatement of drug seeking as well as the long term effect (Figure 2A). During acquisition of heroin self-administration, there was no difference in the total numbers of infusion between the rats that would be infused with propranolol (*N* = 8) and those infused with saline (*N* = 9) [main effect of the training day: F _(9,135)_ = 19.591, *p* < 0.001; main effect of the treatment condition: F _(1,15)_ = 0.055, *p* = 0.817; interaction of training day × treatment condition: F _(9,135)_ = 0.129, *p* = 0.99; Figure 2B]. Similarly, there was no significant group difference in the rate of extinction, as revealed by a two-way repeated-measures ANOVA [main effect of extinction day: F _(9,135)_ = 40.177, *p* < 0.001; main effect of the treatment condition: F _(1,15)_ = 0.507, *p* = 0.488; interaction of extinction day × treatment condition: F _(9,135)_ = 0.327, *p* = 0.965; Figure 2C]. For the reactivation test, there were no group difference of heroin infusions during the retrieval test [main effect of the different nosepokes: F _(1,15)_ = 56.39, *p* < 0.001; main effect of the treatment condition: F _(1,15)_ = 0.435, *p* = 0.520; interaction of test different nosepokes × treatment condition: F _(1,15)_ = 0.124, *p* = 0.730; Figure 2D]. In line with the results from Experiment 1, the immediate post-CS propranolol treatment had significant effects on subsequent cue-induced reinstatement of active nosepoking (i.e., heroin seeking) [main effect of the test condition: F _(1,15)_ = 60.55, *p* < 0.001; main effect of the treatment condition: F _(1, 15)_ = 26.58, *p* < 0.001; interaction of test condition × treatment condition: F _(1,15)_ = 17.55, *p* < 0.001]; A *Post-hoc* test revealed that drug-seeking in the Retrieval + Propranolol group was significantly reduced compared to the Retrieval + Saline group in the cue-induced reinstatement test (*p* < 0.05) (Figure 2E left column), but not inactive nosepokes [main effect of the test condition: F _(1,15)_ = 2.469, *p* = 0.137; main effect of the treatment condition: F _(1, 15)_ = 0.219, *p* = 0.647; interaction of test condition × treatment condition: F _(1,15)_ = 0.159; *p* = 0.696; Figure 2E right column]. Furthermore, a repeated measures (rm)-ANOVA revealed a significant effect of active nosepokes during the cue-induced reinstatement test after prolonged withdrawal [ main effect of the test condition: F _(1,15)_ = 100.9, *p* < 0.001; main effect of the treatment condition: F _(1,15)_ = 25.38, *p* < 0.001; interaction of test condition × treatment condition: F _(1,15)_ = 20.72, *p* < 0.001]; A *post-hoc* revealed that drug-seeking in the Retrieval + Propranolol group was significantly reduced compared to the Retrieval + Saline group during the cue-induced reinstatement test after prolonged withdrawal (*p* < 0.05) (Figure 2F left column) but no significant difference in inactive nosepokes [ main effect of the test condition: F _(1,15)_ = 3.431, *p* = 0.084; main effect of the treatment condition: F _(1,15)_ = 0.293, *p* = 0.597; interaction of test condition × treatment condition: F _(1,15)_ = 0.286, *p* = 0.600; Figure 2F right column]. Thus, these results suggest that the immediate post-CS propranolol treatment inhibits cue-indued heroin seeking and this effect lasts up to 28 days.

### Experiment 3: Intraperitoneal Propranolol Treatment Without Conditioned Stimulus Reactivation Has No Effect on Subsequent Reinstatement Heroin Seeking

**FIGURE 3:**
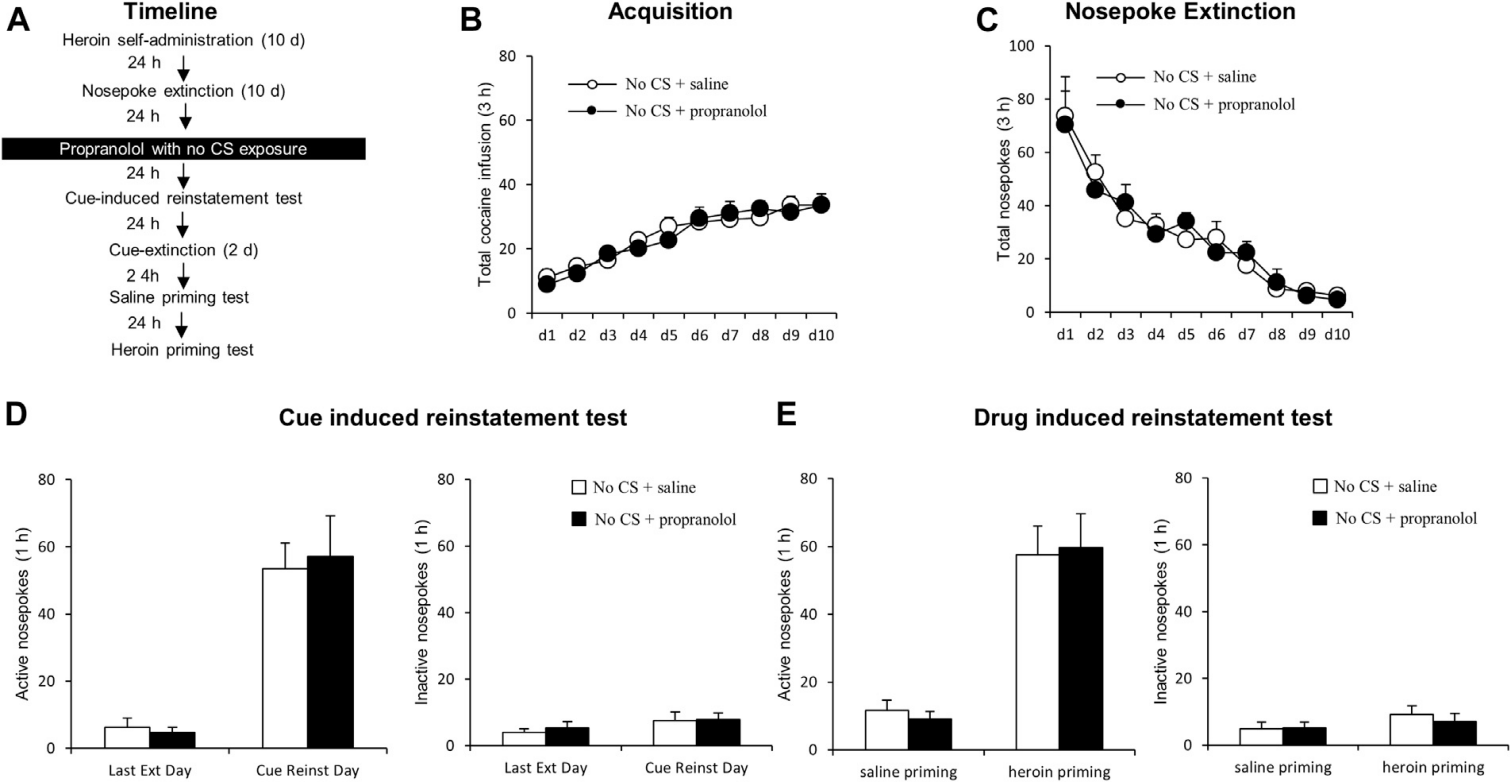
Propranolol treatment without CS reactivation has no effect on subsequent cue-induced and heroin-primed reinstatement of heroin seeking. **(A)** Schematic representation of the experimental procedure. **(B)** Total number of infusions across acquisition of heroin self-administration. **(C)** Total number of active nosepoke responses across extinction sessions. **(D)** Active **(left)** and inactive **(right)** nosepoke responses during the last extinction session and cue-induced reinstatement test. **(E)** Active **(left)** and inactive **(right)** nosepoke responses during the saline-or heroin-primed reinstatement test.

In this experiment, we examined whether the therapeutic effects of propranolol on subsequent heroin seeking is CS reactivation dependent. After heroin self-administration and extinction training, rats received an intraperitoneal injection of propranolol in the absence of a 15-min exposure to the drug cues/context (Figure 3A). No significant group differences were found in heroin self-administration [main effect of the training day: F _(9,108)_ = 31.568, *p* < 0.001; main effect of the treatment condition: F _(1,12)_ = 0.054, *p* = 0.820; interaction of training day × treatment condition: F _(9,108)_ = 0.668, *p* = 0.736; Figure 3B] or nosepoke extinction rates [main effect of the extinction day: F _(9,108)_ = 26.749, *p* < 0.001; main effect of the treatment condition: F _(1,12)_ = 0.001, *p* = 0.972; interaction of extinction day × treatment condition: F _(9,108)_ = 0.384, *p* = 0.941; Figure 3C] between the rats that would be infused with propranolol (*N* = 7) and those infused with normal saline (*N* = 7). During the cue-induced reinstatement test, there were no significant difference in active nosepokes [main effect of the test condition: F _(1,12)_ = 61.35, *p* < 0.001; main effect of the treatment condition: F _(1,12)_ = 0.015, *p* = 0.903; interaction of test condition × treatment condition: F _(1,12)_ = 0.163, *p* = 0.693; Figure 3D, left column] and inactive nosepokes [main effect of the test condition: F _(1,12)_ = 1.863, *p* = 0.197; main effect of the treatment condition: F _(1,12)_ = 0.288, *p* = 0.602; interaction of test condition × treatment condition: F _(1,12)_ = 0.068, *p* = 0.799; Figure 3D, right column] between the groups. During the heroin-primed reinstatement test, there were no significant difference in active nosepokes [main effect of test condition: F _(1,12)_ = 51.668, *p* < 0.001; main effect of treatment condition: F _(1,12)_ = 0.001, *p* = 0.976; interaction of test condition × treatment condition: F _(1,12)_ = 0.123, *p* = 0.732; Figure 3E, left column] and inactive nosepokes [main effect of the test condition: F _(1,12)_ = 2.555, *p* = 0.136; main effect of the treatment condition: F _(1,12)_ = 0.134, *p* = 0.721; interaction of test condition × treatment condition: F _(1,12)_ = 0.399, *p* = 0.539; Figure 3E, right column]. Overall, the results of this experiment indicate that the therapeutic effect of propranolol on heroin seeking depends on the reactivation of drug-related memory and that propranolol alone has no effect on the following cue-induced or heroin-primed reinstatement of heroin seeking.

### Experiment 4: 6 h-Delayed Propranolol Treatment Following Conditioned Stimulus Reactivation has No Effect on Subsequent Reinstatement of Heroin Seeking

**FIGURE 4:**
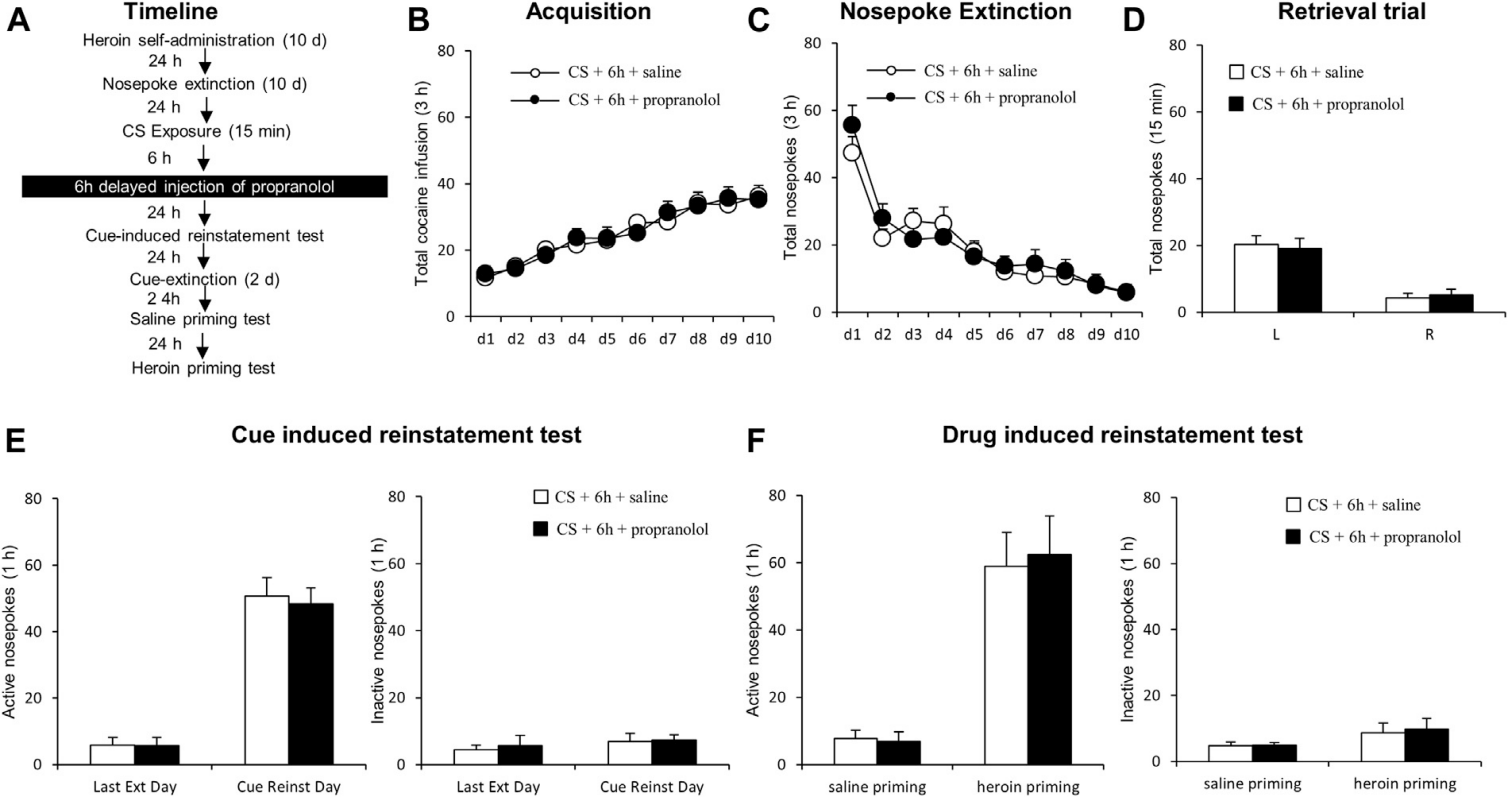
Propranolol treatment 6 h following reactivation has no effect on subsequent reinstatement of heroin-seeking. **(A)** Schematic representation of the experimental procedure. **(B)** Total number of infusions across heroin self-administration sessions. **(C)** Nose-poke responses during the reactivation trial. **(D)** Nose-poke responses during the conditioned reinforcement test. **(E)** Active **(left)** and inactive **(right)** nosepoke responses during the last extinction session and the cue-induced reinstatement test. **(F)** Active **(left)** and inactive **(right)** nosepokes during the saline-or heroin-primed reinstatement test.

Next, we examined whether propranolol administered beyond the sensitive time window of memory reconsolidation would attenuate subsequent heroin seeking (Figure 4A). Groups did not differ in the acquisition of heroin self-administration [propranolol (*N* = 7) *vs.* vehicle (*N* = 7)], as indicated by similar total numbers of heroin infusions [main effect of the training day: F _(9,108)_ = 21.629, *p* < 0.001; main effect of treatment condition: F _(1,12)_ = 0.004, *p* = 0.948; training day interaction × treatment condition: F _(9,108)_ = 0.278, *p* = 0.979; Figure 4B]. Additionally, there was no significant difference in nosepoke extinction rates as revealed by the similar numbers of active and inactive nosepokes across the extinction sessions [main effect of the extinction day: F _(9,108)_ = 26.219, *p* < 0.001; main effect of the treatment condition: F _(1,12)_ = 0.158, *p* = 0.696; interaction of extinction day × treatment condition: F _(9,108)_ = 0.664, *p* = 0.740; Figure 4C]. During the reactivation test, there were no group difference of heroin infusions [main effect of the different nosepokes: F_(1,12)_ = 57.17, *p* < 0.001; main effect of the treatment condition: F _(1,12)_ = 0.007, *p* = 0.937; interaction of different nosepokes × treatment condition: F _(1,12)_ = 0.295, *p* = 0.597; Figure 4D]. Also, no significant difference was observed in active nosepokes during a cue-induced reinstatement test [main effect of the test condition: F _(1, 12)_ = 113.6, *p* < 0.001; main effect of the treatment condition: F _(1, 12)_ = 0.102, *p* = 0.755; interaction of test condition × treatment condition: F _(1, 12)_ = 0.078, *p* = 0.785; Figure 4E, left column], as well as inactive nosepokes [main effect of test condition: F _(1, 12)_ = 0.766, *p* = 0.399; main effect of treatment condition: F _(1,12)_ = 0.144, *p* = 0.711; interaction of test condition × treatment condition: F _(1,12)_ 0.035, *p* = 0.854; Figure 4E, right column]. There is no significant difference in heroin-primed reinstatement of heroin seeking (active nosepoke responses) [main effect of the test condition: F _(1,12)_ = 44.114, *p* < 0.001; main effect of treatment condition: F_(1,12)_ = 0.028, *p* = 0.869; interaction of test condition × treatment condition: F _(1,12)_ = 0.071, *p* = 0.795]; Figure 4 F left column], and inactive nosepokes [main effect of test condition: F _(1,12)_ = 3.112, *p* = 0.103; main effect of treatment condition: F _(1,12)_ = 0.074, *p* = 0.790; interaction of test condition × treatment condition: F _(1,12)_ = 0.029, *p* = 0.867; Figure 4F right column]. The results of this experiment indicate that the effect of propranolol on reconsolidation of heroin reward memory is temporally specific.

## Discussion

Our study examined the effects of propranolol, a non-specific β-adrenoceptor antagonist, on reconsolidation of drug-associated memory using an intravenous heroin self-administration procedure. We found that systemic administration of propranolol immediately, but not 6 h after CS exposure significantly reduced cue-induced or drug-primed reinstatement of heroin seeking, suggesting that propranolol can disrupt heroin memory during memory reconsolidation. Furthermore, we found that the propranolol treatment without retrieval of drug memory had no effect on reinstatement of heroin drug seeking, suggesting that the effects of propranolol on reconsolidation of drug memory are retrieval-dependent. Finally, to test the long-lasting effect of propranolol on memory reconsolidation, the cue-induced reinstatement test was implemented again after 28 days’ prolonged withdrawal. The results revealed that propranolol when administered immediately after CS reactivation reduced heroin seeking even 28 days later, indicating propranolol has potential to permanently reduce heroin seeking. Our findings are in line with previous studies demonstrating that memory reconsolidation can be disrupted after retrieval (Xue et al., 2012; Jian et al., 2014; Luo et al., 2015), and that propranolol can effectively do so when administered within a sensitive time window immediately and with memory retrieval (Przybyslawski et al., 1999; Diergaarde et al., 2006; Xue et al., 2017a). Altogether, the present results indicate that propranolol might be a vital pharmacotherapeutic in relapse prevention.

A large body of literature has shown that cocaine- or opioid CPP memory can be retrieved by drug-conditioned cues or drug itself (Robinson et al., 2011a; Lin et al., 2014; De Carvalho and Takahashi, 2017; Shen et al., 2020; Ding et al., 2021). In the present study, we found that 15 min-CS exposure was sufficient to retrieve consolidated reward memory. Propranolol when employed as a potent amnestic agent, can interfere with drug memory and impair reinstatement of cocaine, nicotine or opioid CPP (Fricks-Gleason and Marshall, 2008; Otis and Mueller, 2011; Robinson et al., 2011b; Xue et al., 2017a) or spontaneous recovery through its antagonistic action on beta-noradrenergic receptors. In the present study we have demonstrated that propranolol can interfere with heroin seeking and relapse by disrupting drug memory during retrieval. However, when implemented with a delay, 6 h after CS exposure, it had no significant therapeutic benefits. Notably, substantial evidence from preclinical studies has shown that drug memories are labile and vulnerable for modification during a putative time window of memory reconsolidation. During memory reconsolidation new proteins are synthesized shortly after memory retrieval and potent protein synthesis inhibitors (e.g., anisomycin) or other agents (e.g., barberine, sulfur dioxide, rapamycin, cannabidiol) can disrupt this process producing long-term effects on drug seeking and relapse (Fuchs et al., 2010; Wells et al., 2011; Wu et al., 2013; Lin et al., 2014; Carvalho and Takahashi, 2016; Shen et al., 2020; Ding et al., 2021).

In this study, cue-induced and drug-primed reinstatement of heroin seeking was impaired by non-specific β-blocker propranolol when administered immediately after CS-retrieval, but not after a 6 hour-delay or without CS reactivation, indicating that the propranolol intervention with memory reconsolidation and relapse requires both temporal specificity and CS retrieval. We also demonstrated that the effects of post CS retrieval propranolol on heroin seeking are long lasting, suggesting relatively permanent interference with drug memory. Indeed, preclinical and clinical studies have demonstrated that propranolol has the ability to erase maladaptive memories associated with nicotine, cocaine, heroin or fear (Fricks-Gleason and Marshall, 2008; Zhao et al., 2011; Xue et al., 2017b; Deng et al., 2020). Thus, there is compelling evidence suggesting that propranolol might have a therapeutic utility in clinical settings and be an attractive candidate for relapse prevention.

In the present study, propranolol that was delivered systematically produced robust effects on drug memory reconsolidation, suggesting that noradrenergic signaling likely controls drug memory reconsolidation. However, the precise noradrenergic mechanisms and neural circuit involved in heroin memory reconsolidation are yet to be determined. One potential target is the amygdala, a brain region involved in associative learning between environment cues and appetitive stimuli (Janak and Tye, 2015). Previous studies revealed that the basolateral amygdala receives the noradrenergic inputs from the nucleus tractus solitarius and locus coeruleus, which are known for their role in memory consolidation and reconsolidation. The amygdala output neurons project to the nucleus accumbens and this pathway is deemed for its critical role in cue-induced drug seeking and drug memory reconsolidation (Otis et al., 2015). In the follow-up studies we plan to systematically identify the neuronal circuitry underlying the noradrenergic signaling involved in drug memory reconsolidation. We intend to target the amygdala and identify its noradrenergic afferents (and its efferents) involved in the process of heroin memory reconsolidation.

Propranolol crosses the blood-brain barrier and targets the β-ARs through noradrenergic signaling system (Pardridge et al., 1983; Westfall and Westfall, 2006); the system is important for memory reconsolidation (Otis et al., 2015). Activation of β-ARs is known to increase neuronal excitability and contribute to synaptic plasticity of the glutamatergic system (Otis et al., 2013; Otis and Mueller, 2017). Thus, neuroplasticity induced by activation of β-ARs is thought to allow drug cues to drive compulsive drug taking and seeking. During memory reconsolidation after CS exposure, reactivated memory comes back to a stable state to be persistently stored, leading to permanent synaptic plasticity (Bonin and De Koninck, 2015; Chu et al., 2019). Propranolol by blocking β noradrenergic receptors appears to take part in memory reconsolidation by interfering with synaptic plasticity. However, specific effects of propranolol on molecules and cellular processes within the noradrenergic system during memory reconsolidation remain to be elucidated.

In the present study, we used an intravenous drug self-administration procedure, which more closely mimics drug abuse in humans (Spealman and Goldberg, 1978; Schindler et al., 2002). We believe that in contrast to a traditional conditioned place preference (CPP) paradigm (Sanchis-Segura and Spanagel, 2006; Tzschentke, 2007), this procedure is more appropriate to study craving and relapse. In fact, in the CPP procedure animals experience drug effects in a passive way and their drug intake are not voluntary, whereas in human addict drug taking and seeking are voluntary and reinforced by drugs and drug cues. In other words, human responses to drugs are contingent on consequences. Therefore, the drug-self-administration procedure more adequately reflects human addiction behaviors when combined with a retrieval trial, it can be used to study memory reconsolidation.

In summary, the present study introduced a potential procedure that combines voluntary drug taking and seeking with a retrieval trial to study the effects of pharmacological intervention on memory reconsolidation and subsequent cue- and drug-induced drug seeking. We have demonstrated that in this paradigm, a non-selective β-Adrenoceptor receptor antagonist, when administered during a sensitive time window after retrieval, can disrupt memory reconsolidation and attenuate subsequent relapse. Overall, our findings suggest that propranolol might be a potential therapeutic candidate for relapse prevention to opioids and treatment of opioid addiction.

## Data Availability

The raw data supporting the conclusions of this article will be made available by the authors, without undue reservation.
